# Barrier Properties of Poly(Propylene Cyclohexanedicarboxylate) Random Eco-Friendly Copolyesters

**DOI:** 10.3390/polym10050502

**Published:** 2018-05-05

**Authors:** Valentina Siracusa, Laura Genovese, Carlo Ingrao, Andrea Munari, Nadia Lotti

**Affiliations:** 1 Department of Chemical Science, University of Catania, Viale A. Doria 6, 95125 Catania (CT), Italy; ing.carloingrao@gmail.com; 2 Department of Civil, Chemical, Environmental and Materials Engineering, University of Bologna, Via Terracini 28, 40131 Bologna (BO), Italy; laura.genovese@unibo.it (L.G.); andrea.munari@unibo.it (A.M.); nadia.lotti@unibo.it (N.L.)

**Keywords:** biodegradable polymers, Poly(propylene 1,4-cyclohexanedicarboxylate), random copolymers, gas barrier properties, food packaging, eco-friendly copolyesters, food simulants, relative humidity

## Abstract

Random copolymers of poly(propylene 1,4-cyclohexanedicarboxylate) containing different amounts of neopentyl glycol sub-unit were investigated from the gas barrier point of view at the standard temperature of analysis (23 °C) with respect to the three main gases used in food packaging field: N_2_, O_2_, and CO_2_. The effect of temperature was also evaluated, considering two temperatures close to the *T*_g_ sample (8 and 15 °C) and two above *T*_g_ (30 and 38 °C). Barrier performances were checked after food contact simulants and in different relative humidity (RH) environments obtained with two saturated saline solutions (Standard Atmosphere, 23 °C, 85% of RH, with saturated KCl solution; Tropical Climate, 38 °C, 90% RH, with saturated KNO_3_ solution). The results obtained were compared to those of untreated film, which was used as a reference. The relationships between the gas transmission rate, the diffusion coefficients, the solubility, and the copolymer composition were established. The results highlighted a correlation between barrier performance and copolymer composition and the applied treatment. In particular, copolymerization did not cause a worsening of the barrier properties, whereas the different treatments differently influenced the gas barrier behavior, depending on the chemical polymer structure. After treatment, Fourier transform infrared analysis confirmed the chemical stability of these copolymers. Films were transparent, with a light yellowish color, slightly more intense after all treatments.

## 1. Introduction

Plastic packaging, particularly that used for food packaging, accounts for a large proportion of the total polymer production due to a combination of several favorable factors such as being lightweight, flexible, strong, stable, impermeable, and easy to sterilize. Due to their high versatility and safety, plastics are used for fresh meat, beverages, oils and sauces, fruit and vegetables, yoghurt, fish, essentially for all kinds of food. Most importantly, food packaging must guarantee food conservation and preservation for long periods, simultaneously reducing time wastage and the use of preservatives. Prolongation of the shelf-life results in considerable savings in terms of money, material consumption, and food waste.

Shelf-life has been defined many times, but no definition has been regulated. The European Commission Regulation (EC) No. 2073/2005 provided the following definition: shelf-life is the time frame corresponding to the period preceding the “use by” or the “minimum durability date”, with “use by” and “minimum durability date” taken from Art. 9 and 10 of Directive 200/13/EC). Food shelf-life is governed by several factors: (1) the intrinsic food characteristics, like pH, water activity, fat content, nutrient, respiration rate, and biological structure; (2) environmental influences such as temperature, relative humidity, and gas surrounding (permeability factor); and (3) the type of packaging. Control over these three parameters contributes to prolonging, or at least maintaining food quality during the “use by” time.

Moreover, food deterioration can occur via chemical, biochemical, physical, and microbiological attack. Several materials, such as paper, glass, or metal, have been used for food preservation. Only in the last century have plastics appeared, being the current most used material for food packaging. Plastics must fulfil strict requirements to be used for this application. In particular, they have to be “passive”, “active”, and/or “intelligent”. “Passive” means that packaging must not interact with food; plastic packaging must maintain the same physical, chemical, mechanical, and permeability behavior during the food’s entire shelf life, and no migration of monomers or additives can occur from packaging to food. Food organoleptic properties, including smell, taste, color, and texture, must also remain unaltered. “Active” implies that packaging should perform an active role when used for food preservation (modify its properties), remaining “passive” in order to ensure food shelf life extension, to improve safety and sensory properties, and to maintain the food quality [1]. To this purpose, the incorporation of additives could represent a solution to create an “active packaging”. So far, many additives have been introduced in the market: oxygen scavengers, CO_2_, O_2_, C_2_H_4_, and moisture absorbers, antimicrobial agents, etc. Lastly, “intelligent” means that plastic has to be able to communicate with food inside the package and the surroundings outside the package. Although “passive” and “active” represent intrinsic material properties, “intelligence” is an added characteristic, readable by the consumer [2]. 

The huge amount of plastic packaging produced annually quickly becomes, significantly contributing to the pollution of aquatic and terrestrial environments. Therefore, growing environmental awareness is creating a need for package film to be eco-friendly [3]. In addition to recycling used packages, the development of materials with biodegradability and/or compostability attributes would reduce municipal solid waste [4]. As a result, biodegradability is not only a functional requirement, but is also crucial from an environmental point of view [3], especially considering that the bioplastics industry is growing at a rate of more than 20% per year [5].

With the aim of broadening the spectrum of bioplastics used in this field, several research groups have been focusing their efforts on developing new bioplastics, compounds, and master batches. Until now, only a limited number of these materials have been made available on the market for food packaging applications, with most common being aliphatic polyesters, above all Poly(lactic acid) (PLA), starch, and cellulose [4,6,7]. Aliphatic polyesters can be considered very competitive, as most are biodegradable and bio-based. Some of the monomers used for their production, such as succinic acid, adipic acid, 1,3-propanediol, 1,4-butanediol, lactic acid, and γ-butyrolactone, can be obtained from both petroleum resources and renewable resources [8]. Within this class, polyesters with cycloaliphatic units like poly(alkylene 1,4-cyclohexanedicarboxylate)s offer several advantages: excellent tensile strength, stiffness and impact properties, high thermal stability, and easily attackable by microorganisms that allow these materials to be biodegradable. Furthermore, most of poly(alkylene 1,4-cyclohexanedicarboxylate)s are biodegradable and bio-based materials. The monomer 1,4-cyclohexane dicarboxylic acid can be obtained from bio-based terephthalic acid starting from limonene and other terpenes [8]. Our research group synthesized a poly(butylene 1,4-cyclohexane dicarboxylate) homopolymer as well as some of its random and block copolymers with the aim of evaluating the potential of these new materials to be used for eco-friendly food packaging. The barrier performances appeared to be promising, being superior compared with some traditional fossil-based plastics, strictly correlated to material chemical structure [9,10,11].

Poly(propylene 1,4-cyclohexanedicarboxylate) (PPCE) and its random copolymers containing different amounts of neopentyl glycol sub-units (P(PCExNCEy) have been synthesized and characterized from the molecular, thermal, structural, and mechanical points of view [12]. These new polyesters represent a new class of bio-based and biodegradable ecofriendly materials with the potential for use in food packaging applications.

Copolymerization is a useful tool for obtaining materials with well-tailored properties for the final desired application [13,14]. Using this strategy, synthetizing a new class of materials with improved characteristics is possible without compromising the pre-existing satisfying characteristics. 

As the gas barrier behavior is fundamental in food packaging applications in order to select the best material for prolonging food shelf life, while maintaining the safety and quality of the packed food throughout the storage period, our research work focused on the study of such properties in different situations for PPCE and P(PCExNCEy) random copolymers. The gas barrier behavior was first analyzed in standard conditions with the three main gases, nitrogen, oxygen, and carbon dioxide (N_2_, O_2_ and CO_2_), used with the modified atmosphere packaging technique (MAP). The permeation mechanism was studied in the temperature range of 8 to 38 °C in order to understand the influence of temperature and to calculate the activation energy of the permeation process. Then, a different moisture environment was considered, simulating storage under standard atmosphere (85% relative humidity) and in tropical atmosphere (90% relative humidity). Lastly, food contact was mimicked, with the use of four food simulant liquids, following the guideline provided by the European Regulation for packaging in contact with food. The correlation between chemical polymer structure and barrier properties was determined to establish structure-property relationships, which are of fundamental importance for evaluating the suitability of a certain material for a specific application.

## 2. Materials and Methods 

### 2.1. Materials

1,4-dimethylcyclohexanedicarboxylate (DMCE), containing 99% trans isomer (TCI Europe, Zwijndrecht, Belgium), 1,3-propanediol (1,3-PD), neopentyl glycol (NPG), and titanium tetrabutoxide (Ti(OBu)_4_) (Sigma Aldrich, Milan, Italy) were used as supplied, without any preliminary purification. Only the catalyst Ti(OBu)_4_ was distilled before use.

### 2.2. Polymer Synthesis, Film Preparation, and Thickness Determination

Poly(propylene cyclohexanedicarboxylate) (PPCE) and poly(propylene/neopenthyl glycol cyclohexanedicarboxylate) random copolymers (P(PCExNCEy)) were synthesized according to the procedure reported by Genovese et al. [12]. The polymerization was performed starting from DMCE and 1,3-PD for the homopolymer, and different ratios of PD/NPG for the copolymers, with 40% mol excess glycol with respect to dimethylester. A total of 150 ppm of Ti(OBu)_4_ per g of polymer were used. The polymers were prepared according to the two-stage polymerization procedure. In the first step, the temperature was raised to 180 °C and kept constant for about 120 min, until more than 90% of the methanol was distilled off. In the second step, the pressure was reduced to about 0.1 mbar to facilitate the removal of the residual methanol and the excess glycol. The temperature was increased to 240 °C and the polymerization was performed for about 180 min. Temperature and torque were continuously recorded during the polymerization. The syntheses were performed in a 250 mL glass reactor under continuous stirring in a thermostated silicon oil bath.

**Figure 1:**
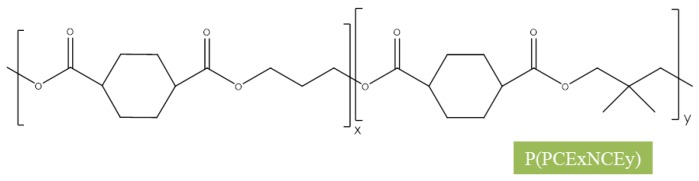
Chemical formula of neopentyl glycol sub-units P(PCExNCEy) random copolyesters.

The obtained copolymers were indicated as P(PCExNCEy) with x and y of the mol % of propylene 1,4-cyclohexanedicarboxylate (PCE) and neopenthyl glycol 1,4-cyclohexanedicarboxylate (NCE) co-monomeric units, respectively. For simplicity, the chemical formula of the synthesized copolyesters is shown in Figure 1.

Films of P(PCExNCEy) were obtained by hot pressing in a hydraulic press (Carver Inc., Wabash, IN, USA). The powder was placed between two sheets of Teflon for 2 min at a temperature *T* equal to *T*_m_ + 40 °C, with *T*_m_ being the fusion temperature determined by calorimetric experiments. The films were cooled directly in the press until reaching room temperature by running water. 

Before completing the analyses, the films were maintained at room temperature for at least three weeks to reach crystallinity equilibrium. 

The film thickness was determined using the Sample Thickness Tester DM-G (Brugger Feinmechanik GmbH, Munich, Germany), consisting of a digital indicator (Digital Dial Indicator) connected to a PC. The reading was made twice per second (the tool automatically performs at least three readings), measuring a minimum, a maximum, and an average value. The thickness value is expressed in μm and the measured values range from 240 to 310 μm, with a resolution of 0.001 μm. The reported results represent the mean value thickness of the three experimental tests run at 10 different points on the polymer film surface at room temperature.

### 2.3. Gas Transport Measurements

The determination of the gas barrier behavior was performed using a manometric method, using a Permeance Testing Device, type GDP-C (Brugger Feinmechanik GmbH, Munich, Germany), according to ASTM 1434-82 (Standard test Method for Determining Gas Permeability Characteristics of Plastic Film and Sheeting), DIN 53 536 in compliance with ISO/DIS 15 105-1, and according to the Gas Permeability Testing Manual [15].

After a preliminary high vacuum desorption of the system, the upper chamber was filled with the gas test at ambient pressure. A pressure transducer, set in the chamber below the film, recorded the increasing gas pressure as a function of time. The gas transmission rate (GTR, expressed in cm^3^·m^−2^·d^−1^·bar^−1^) was determined by considering the increase in pressure in relation to the time and the volume of the device. The pressure was given by the instrument in bar units. To obtain the data value in kPa, the primary SI units, we used the following correction factor: 1 bar = 10 kPa, according to NIST special publication 811 [16]. Films were analyzed at 23 °C (the standard temperature of analysis), at 8 and 15 °C (around *T*_g_), and 30 and 38 °C (above *T*_g_), with a food grade gas stream of 100 cm^3^·min^−1^ and a 0% gas RH. Gas transmission measurements were performed at least in triplicate and the mean value is presented. Method A was used for the analysis, as previously reported in the literature [15,17] with the evacuation of the top and bottom chambers. The sample temperature was set by an external thermostat HAAKE-Circulator DC10-K15 type (Thermo Fisher Scientific, Waltham, MA, USA). 

The experiments were performed in triplicate and the results are presented as the average ± standard deviation. 

The transport phenomena background is well described in the literature, with a full description of the mathematical equation and interpretation [18,19].

### 2.4. Relative Humidity Solution

According to the procedure reported in the Gas Permeability Testing Manual [15], the analyses were performed at different relative humidity (RH) obtained with several saturated saline solutions. In particular, we performed the analyses at Standard Atmosphere, which is 23 °C, 85% RH, with a saturated KCl solution, and at Tropical Climate, which is 38 °C, 90% RH, with a saturated KNO_3_ solution.

The values of the relative humidity for the saline solutions were obtained from DIN 53 122 part 2. A glass-fiber round filter humidified with the desired saturated saline solution was inserted in the humid part of the top permeation cell. Method C was used, with gas flow blocked from the test specimen during evacuation. Using this method, the test gas was humidified inside the permeation cell. This method evacuates only the area of the bottom part of the sample. On the top part of the test specimen, filled with the humidified gas, normal ambient pressure was applied. 

### 2.5. Simulant Liquids

The food contact simulation was performed in accordance with *EU Regulations No. 10/2011 on plastic materials and articles intended to come into contact with food* [20]. Four solutions were used as food simulants: (1) Simulant A, Ethanol 10% (*v*/*v*), 10 days, 40 °C; (2) Simulant B, Acetic acid 3% (*v*/*v*), 10 days, 40 °C; (3) Simulant C, Ethanol 20% (*v*/*v*), 10 days, 40 °C; and (4) Simulant D1, Ethanol 50% (*v*/*v*), 10 days, 40 °C.

Measurements were collected from a completely immersed 12 cm × 12 cm film specimen. A total of 200 mL of simulant were placed into 400 mL glass flasks containing the film sample and the flasks were then covered with caps. Samples were placed in a stove (Universalschrank UF110, Memmert GmbH + Co. KG, Schwabach, Germany). After the assay time elapsed, the specimens were removed from the flasks, washed with distilled water twice, and dried with blotting paper. Before analysis, the films were kept at room temperature, in dry ambient conditions for at least two weeks. The samples were tested in triplicate.

### 2.6. FTIR Spectroscopic Analysis

The Fourier transform infrared (FTIR)/ATR spectra were recorded on sample films by a Perkin-Elmer-1725-X Spectrophotometer (Labexchange Group, Burlandingen, Germany). Spectra were recorded from 4000 to 600 cm^−1^ with a resolution of 4.0 cm^−1^. The results are an average of 10 experimental tests, run on 10 different sample points to test the results’ reproducibility. Sixty-four scans were recorded on each sample. The experiments were performed at room temperature, directly on the samples, without any preliminary treatments.

### 2.7. Color Evaluation

The color of the film samples was measured using a HunterLab ColorFlex EZ 45/0° color spectro-photometer (Reston, VA, USA) with D65 illuminant and 10° observer according to ASTM E308. Measurements were recorded using CIE Lab scale. The instrument was calibrated with a black and white tile before the measurements. Results were expressed as L* (lightness), a* (red/green), and b*(yellow/blue) parameters. The total color difference (Δ*E*) was calculated using the following equation: Δ*E* = [(Δ*L*)^2^ + (Δ*a*)^2^ + (Δ*b*)^2^ ]^0.5^, where Δ*L*, Δ*a*, and Δ*b* are the differentials between a sample color parameter (L*, a*, and b*) and the color parameter of a standard white plate used as the film background (*L*’ = 66.39, *a*’ = −0.74, and *b*’ = 1.25). Chromaticity C* = [(a*)^2^ + (b*)^2^]^0.5^ and hue angle *h*_ab_ = [arctan (b*/a*)/2π] 360, were calculated, as previously reported in the literature [21,22,23]. Measurements were recorded in triplicate at random positions over the film surface. Average values are reported.

### 2.8. Molecular Weight Determination

Molecular weights were evaluated by gel-permeation chromatography (GPC) at 30 °C using a 1100 high performance liquid chromatography (HPLC) system (Agilent Technologies, Santa Clara, CA, U.S.) equipped with PLgel μmeter MiniMIX-C column (Agilent Technologies). A refractive index was used as the detector. Chloroform was used as the eluent with a 0.3 mL/min flow and sample concentrations of about 2 mg/mL. A molecular weight calibration curve was obtained with polystyrene standards in the range of 2000 to 100,000 g/mol.

### 2.9. Statistical Analysis

One-way analysis of variance (ANOVA) and testing of the mean comparisons according to Fisher’s least significant differences (LSDs) was applied to the obtained results, with a level of significance of 0.05. The statistical package STS Statistical for Windows, version 6.0 (Statsoft Inc., Tulsa, OK, USA) was used. Values are given as mean ± SD of 3 replicates.

## 3. Results and Discussion

### 3.1. Molecular Characterization

All samples were synthesized and characterized as previously reported [12]. Some of the characterization data are provided in Table 1. These data are useful for the future interpretation of gas barrier behavior.

The synthetized polyesters appeared as opaque and slightly yellow powders, and were characterized by high and comparable molecular weight and by a real copolymer molar composition very close to the feed copolymer. The chemical structure and the real copolymer composition were determined by ^1^H NMR analysis. By NMR, it was possible to calculate the ratio between the *trans* and *cis* forms of the 1,4-cyclohexylene ring present in the DMCE molecule. In particular, less than 5% of the *cis* form was evicted after the polymerization process. All the copolymers showed a high thermal stability, comparable to that of the PPCE homopolymer, due to the presence of a cycloaliphatic ring, which is more thermally stable than a benzene ring [12]. The glass transition temperature was not always evident. A slight increase in *T*_g_ as the amount of NCE co-units increased was observed, in agreement with previously obtained results [24,25,26]. However, all the studied polymers were in the rubbery state at room temperature. Both calorimetric and diffractometric analyses showed a modest reduction in the degree of polymer crystallinity by copolymerization, suggesting the partial inclusion of NCE co-units into the PPCE crystal lattice (partial co-crystallization) [12]. 

### 3.2. Barrier Properties

Carbon dioxide, oxygen, and nitrogen are the main gases used in the food packaging field, especially in Modified Atmosphere Packaging (MAP) technology. These gases may transfer through the packaging wall, continuously influencing the food shelf life as well as food safety and quality. Therefore, gas permeation studies are fundamental to understand and find the best packaging solution, avoiding food damage and losses. As reported in the literature, several parameters can be considered that affect the final barrier performance of a polymeric film, such as temperature, thickness, polymer chemical structure, moisture environment, kind of food in contact with the package, etc. [2].

The samples were analyzed at 23 °C (standard condition) in the range of 8 to 38 °C, in order to study the temperature-permeability dependence, in two different moisture ambient environments, simulating the standard atmosphere (23 °C, 85% RH obtained with KCl saturated saline solution) and a tropical atmosphere (at 38 °C, 90% of RH obtained with KNO_3_ saturated saline solution). The barrier performances were also evaluated after food simulant contact. The results were compared to untreated samples to study the effect of these treatments.

#### 3.2.1. Barrier Properties under the Standard Condition

Permeability was expressed as GTR (cm^3^·cm/m^2^·d·atm), normalized for sample thickness. To convert this unit to others reported in the literature, the converting factors reported by Robertson were used [2]. The theoretical models, the permeation process mechanism, and the permeability coefficients were previously described [18,27].

**Figure 2:**
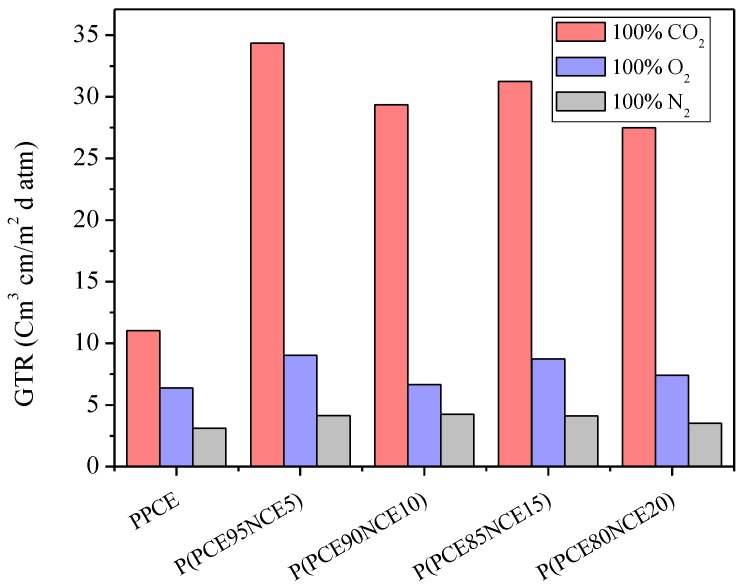
Gas transmission rate at 23 °C for the nitrogen (N_2_), oxygen (O_2_), and carbon dioxide (CO_2_) gas tests.

The gas transmission rate data, recorded for all the samples under study, are reported in Figure 2. Measures were recorded at 23 °C with the three main gases, N_2_, O_2_, and CO_2_, used for food packaging, especially for the modified atmosphere packaging technique (MAP). As previously reported [28], O_2_ is responsible for the food respiration rate. By decreasing O_2_, it is possible to reduce the enzymatic degradation, extending the food shelf-life. However, if the O_2_ amount becomes too low, off-flower and off-odors could be produced, leading to food tissue deterioration. CO_2_, as explained by Farber [29], confers antimicrobial behavior to the food packed, whereas N_2_ is used as an inert gas to complete the inside package atmosphere and to prevent film collapse. The best mix of these gases is fundamental to preserving food quality and safety during the entire storage time. In Table 2, the *S*, *D*, and *t*_L_ data recorded with the CO_2_ gas test are reported. It was not possible to detect the same parameters with the N_2_ and O_2_ gas tests because of the inability to fit the slope of the linear portion of the GTR curves [30]. Perm-selectivity ratios are also reported in Table 2.

As observed from the experimental data, CO_2_ was more permeable than O_2_ and N_2_ for all the samples under study, as reported for other similar polymers previously investigated [28,31], due to the diffusivity drop and solubility increase with decreasing permeant size (molecular diameter of CO_2_ 3.4 Å, oxygen molecular diameter 3.1 Å, and nitrogen molecule diameter 2.0 Å, respectively) [2]. Copolymerization appeared to affect slightly the permeation behavior, which changed with copolymer composition. As previously reported [12], an insertion of bigger NCE units into the PPCE crystal cell was observed by X-ray diffraction analysis. Consequently, the cocrystallization supported the modest decrease in the degree of crystallinity, as well as the reduction in the melting temperature. The introduction of NCE co-units along PPCE macromolecular chains caused a worsening of the barrier performance, particularly pronounced in the case of the most permeable gas, carbon dioxide. As an example, for a copolymer containing the highest amount of comonomeric units (P(PCE80NCE20)), the ratio of GTR to carbon dioxide is 2.5 times higher than that of the PPCE homopolymer, whereas GTR to oxygen is of the same order of magnitude. In general, in the case of the oxygen and nitrogen gas tests, the GTRs of the copolymers are only slightly higher than that of the PPCE homopolymer. This result is analogous to the one we previously found by investigating Poly(L-lactic acid) (PLLA)-based triblock copolymers, containing poly(butylene/neopentyl glycol succinate) random copolymers as central soft block [32]. The short ramifications (methyl groups) exert an obstacle effect toward the small permeant molecules, such as oxygen and nitrogen; their effect being practically negligible in the case of large CO_2_ molecules. Hydrophobic side alkyl groups contribute to reducing the solubility of carbon dioxide in the polymer matrix, with a consequent increase in GTR (see value reported in Table 2). On the contrary, the diffusion coefficient of copolymers appeared to be higher, explaining the higher values of GTR in the CO_2_ gas test. The effect of copolymer composition is not as clear; in fact, an alternating trend was observed with increasing the NCE co-unit amount. As mentioned above, all the copolymers at room temperature were above their glass transition temperature and semicrystalline with a similar crystallinity degree. The polymer polydispersity index (*D*_index_) is among the factors affecting permeability behavior: the lower the *D*_index_, the better the barrier performances. As seen from the data reported in Table 1, P(PCE95NCE5) and P(PCE85NCE15) had higher D_index_ values with respect to P(PCE90NCE10) and P(PCE80NCE10) samples, explaining the observed GTR trend (Figure 2).

The *D*, *S*, and t_L_ parameters were previously fully described by Siracusa et al. [18]. As can be observed from the data reported in Table 2, with respect to the PPCE homopolymer, the *S* value decreased, meaning a lower CO_2_ solubility within the matrix, whereas the *D* value increased, meaning more diffusivity of the gas molecules through the films. Consequently, the t_L_ value was lower than the homopolymer value, due to the less time being needed to reach the permeability steady-state. The GTR rate increase recorded for the copolymers is a direct consequence of this behavior. 

Perm-selectivity values, which represent the permeability ratio of different permeants, are also reported in Table 2. Those values are useful because they allow the calculation of the unknown GTR date, knowing the GTR value of another gas. As reported in the literature [33], for many polymers the N_2_:O_2_:CO_2_ is in the range of 1:4:16. Our results are very different than those tabulated in the literature, demonstrating that using these values for calculation would not be appropriate. As previously demonstrated [11,31], those ratios are not constant, but depend on the chemical structure, temperature, moisture, and kind of simulant in contact with food.

**Figure 3:**
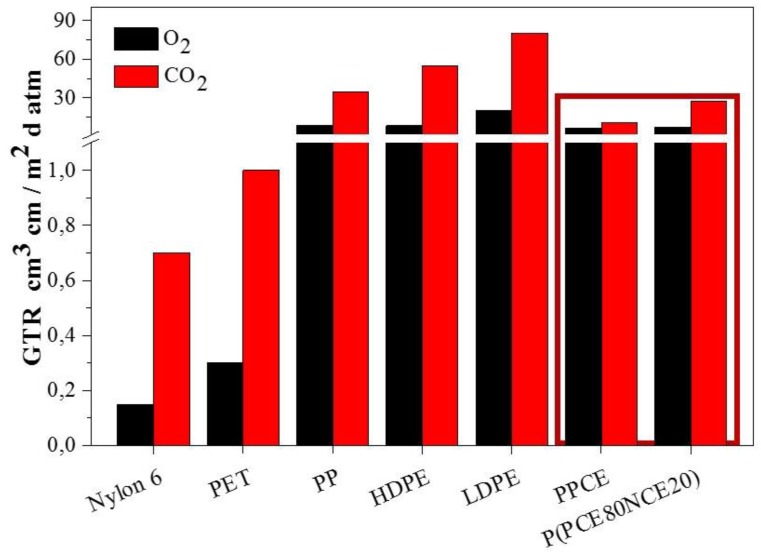
Gas transmission rate of O_2_ and CO_2_ gases for PPCE and P(PCE80NCE20) and some common petrochemical-based polymeric packaging materials [13].

Lastly, PPCE homopolymer and P(PCE80NCE20) copolymer were compared with some common petrochemical-based polymeric packaging materials (Figure 3). Both samples exhibit very good performance in terms of barrier properties against CO_2_ and O_2_, being worse than Nylon6 and polyethylene terephthalate (PET). Whilst this comparison is far from being exhaustive, it can be considered meaningful to highlight the potential of PPCE and PPCE-based copolymers for use as high barrier films.

#### 3.2.2. Activation Energy of Gas Transport Process

**Figure 4:**
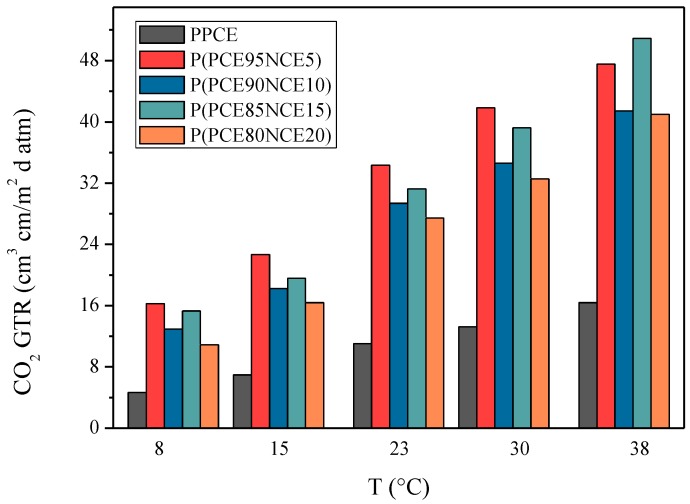
CO_2_-GTR at different temperatures for PPCE and P(PCExNCEy) copolymers.

Temperature is one of the most important parameters affecting food respiration rate. To understand the behavior of polymer membranes in terms of gas permeation, the effect of temperature on permeation behavior has to be evaluated [26]. To establish a correlation with temperature and calculate the activation energies of the permeation processes, the barrier behavior was investigated in the range of 8 to 38 °C. This temperature range was chosen considering all possible temperature scenarios, from food preservation (lower temperatures) to food handling (higher temperatures). Limited information is available about film permeability properties at different storage temperatures. As can be observed in Figure 4, an increase in the GTR was recorded for all samples, being less evident for the PPCE homopolymer.

**Figure 5:**
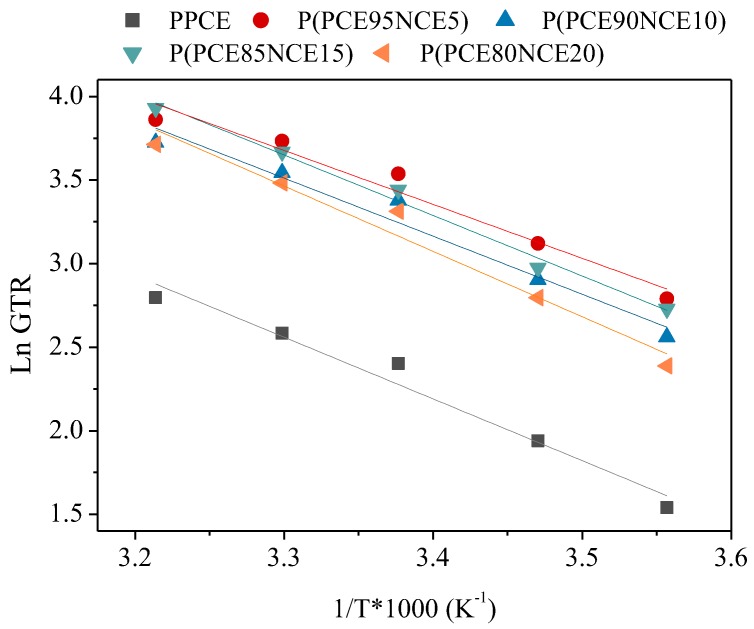
Arrhenius plot of GTR for PPCE and P(PCExNCEy) copolymers.

The CO_2_ gas transfer rate was still higher than those of O_2_ and N_2_ (data not reported). The behavior confirmed the previously observed gas barrier trend. S, D, and *t*_L_ followed the theoretical behavior. By increasing the temperature, S decreased, D increased, and *t*_L_ decreased. The S value, correlated with gas solubility, indicated a reduction in CO_2_ interaction with the polymer matrix. Consequently, *D*, which is correlated to the kinetic parameter, increased and the *t*_L_ decreased with increasing temperature. To describe the permeation dependence on the temperature, the Arrehenius model was used. The activation energy for the gas transmission rate (*E*_GTR_), the specific heat of gas solution (*H*_S_), and gas diffusivity (*E*_D_) processes were calculated using the mathematical relationships reported in the literature [17,33,34]. The activation energy was calculated from the slope (–*E*_a_/*R*) of the straight line obtained plotting ln(GTR) as a function of 1/T for all the samples under investigation (Figure 5), where *R* is the universal gas constant, equal to 8.314 J/mol K. From Figure 5, the experimental data are well-fitted by an Arrhenius-type equation; a high value of the regression coefficient (*R*^2^) was obtained from the fitting. In Table 3, the gas transmission rate data in the temperature range of 8 to 38 °C with CO_2_ gas test are reported. No data were obtained for the O_2_ and N_2_ gas tests because it was not possible to fit the slope of the linear portion of the GTR curves [30].

In general, the activation energy values for gases that migrate through a polymer membrane range from 12 to 63 KJ/mol [34]. The activation energies for the samples under investigation ranged from 27 to 33 KJ/mol, being very similar to those reported by other authors PET, Polyethylene furanoate (PEF), and poly(neopentyl glycol furanoate) (PNF) [19,26,35]. As reported in the literature [36,37,38], high activation energy implies more sensitivity to temperature deviation. Whereas the permeation process is characterized by a good correlation with the temperature variation, the sorption and diffusion process shows consistent deviation due to the chemical composition of the polymers. In general, the solubility decreased with temperature and it is the parameter correlated with the polymer composition. The recorded trend is a confirmation that the gas interacts differently with the polymer matrix. From low to high temperatures, the ln*S* value fluctuated as did the ln*D* values.

#### 3.2.3. Barrier Properties at Different Relative Humidity 

**Figure 6:**
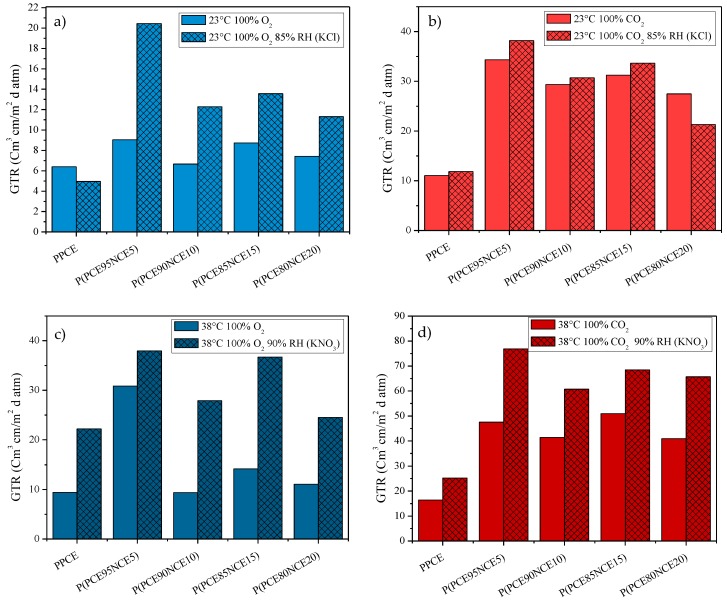
(**a**) O_2_-GTR and (**b**) CO_2_-GTR at 23 °C with 85% relative humidity (Standard Atmosphere). (**c**) O_2_-GTR and (**d**) CO_2_-GTR at 38 °C, 90% relative humidity (Tropical Atmosphere).

Materials characterized by good barrier properties in dry ambient conditions can perform differently when tested in different environments, for example in water. As reported in the literature [39], in the case of low barrier films, the medium reduces the gas permeation rate, whereas for higher barrier materials, like poly(vinylidene-chloride) (PVDG), the influence of the environment on the permeation process is almost undetectable. The GTR results for the PPCE and P(PCExNCEy) samples at different relative humidity are reported in Figure 6.

As reported in the literature [40,41,42], plasticization and swelling phenomena can occur in moist ambient environments. The hydrogen bonds and/or dipole–dipole interactions between water and the polar side of the polymer chains are responsible for this behavior. In particular, due to the water interaction, small network fragments are lost, promoting the transfer of the gas throughout the film. The effects of these phenomena become more intense as the relative humidity and temperature increase. As shown in Figure 6, an increase in the GTR was recorded with increasing RH due to the presence of ester polar groups. In particular, for the sake of simplicity, Table 4 reports the percentage of increase or decrease with respect to the results recorded on the samples without treatments.

A progressive increase in the gas transmission rate was recorded at increasing RH and temperature. In particular, a considerable increase was recorded at 38 °C from 0% to 90% RH for both gases. In general, barrier properties worsened at higher relative humidity, highlighting that the water played an important role in the gas transport process in a wet polymer matrix. The permeability of wet polymers did not follow the same trend as the dry samples. In some cases, the O_2_-GTR was higher than the CO_2_-GTR and vice versa, highlighting that several different factors play a role in the gas barrier behavior. The concomitant action of these factors prevents the establishment of a clear correlation between the chemical structure and barrier behavior under different environmental conditions.

Regardless, these results are important because they account for the strong gas-water-polymer matrix interactions always present for the real conditions during the use of the packaging.

#### 3.2.4. Barrier after Food Simulant Contact

**Figure 7:**
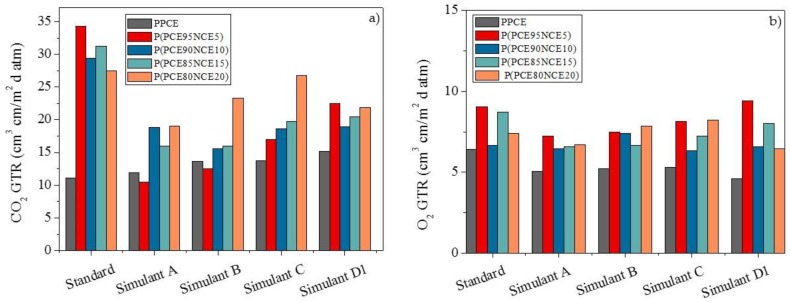
(**a**) CO_2_-GTR and (**b**) O_2_-GTR after food simulant contact for PPCE homopolymer and P(PCE_x_NCE_y_) copolymers.

The GTR data recorded after food simulant contact with the CO_2_ and O_2_ gas tests are reported in Figure 7, together with those of untreated samples added for comparison.

When polymer matrixes are used for food packaging applications, and the polymer packages are consequently placed in contact with food, several scenarios must be considered due to the different characteristics of food.

In particular, as reported in the literature [43,44], food can be aqueous, acid, containing oil/fat, oily or fatty, alcoholic, or low moisture content solid food. To understand the behavior of the materials under study, the worst of the foreseeable conditions were chosen in terms of contact time and temperature (Table 1, Table 2 and Table 3 of the E.U. Regulation) [20]. To perform the analysis, the test number OM2 was chosen in order to analyze a large spectra of food packaging scenarios with a contact time of 10 days at 40 °C. This test is used to analyze the packaging behavior for any long-term food storage at room temperature or below, at heating up to 70 °C for 2 h, and at heating up to 100 °C for up to 15 min. This test also covers the food contact conditions covered by test numbers OM1 and OM3. In particular, food simulants A, B, C, and D1 were chosen. According to the E.U. regulation: (1) simulant A, B, and C simulate the contact of packaging plastic material with food characterized by a hydrophilic character, able to extract hydrophilic substances; (2) simulant B is indicated for food with a pH below 4.5; (3) simulant C is indicated for alcoholic food with an alcohol content up to 20% and for food containing relevant amounts of organic ingredients that render the food more lipophilic; and (4) simulant D1 is indicated for food with a lipophilic character and able to extract lipophilic substances, that mimic alcoholic food with an alcohol content above 20%, and oil in water solution. 

For simplicity, the percentages of GTR increase or decrease (±, %) with respect to the results recorded on the untreated samples are reported in Table 5.

When CO_2_ was used as the test gas, an increase in the GTR value was recorded for the PPCE homopolymer, ranging from 8 to 37%, with the highest value for simulant D1. Interestingly, for all the copolymers investigated, a consistent decrease in the GTR values was recorded, indicating the great stability of these materials when in contact with the food simulants under the worst conditions. In the case of the O_2_ gas test, a general stability, with a light decrease in the GTR values, was also recorded. This behavior was supported by the *D*, *S*, and *t*_L_ data (data not reported). The S value increased after food simulant contact, indicating a higher compatibility of the gas with the polymer. Consequently, the D value decreased, due to the lower speed of the gas molecules moving through the polymer membrane. Thus, the time to attain the steady-state was longer, due to requiring more time for the gas molecules to homogeneously arrange inside the polymer. Due to the influence of several parameters in the permeability process, clear correlation with polymer chemical structure could not be confirmed [2]. Further analyses are in progress to correlate the changes in gas permeation behavior to the chemical and morphological characteristics (change in the crystalline/amorphous ratio after treatments).

### 3.3. FTIR Characterization, Molecular Weigt Determination and Color Evaluation

#### 3.3.1. FTIR Characterization and Molecular Weight Determination

FTIR spectra were recorded for each sample to investigate the change in the chemical structure due to the different treatments. The principal absorption bands for all films are summarized in Table 6. From the spectra, no substantial changes were recorded after each treatment. The main peaks were still present with a small change in the band intensity by increasing the NCE co-unit content due to the presence of –CH_3_ pendant groups. A shift of no more than ±10 cm^−1^ was recorded with respect to the untreated samples. The shift was more evident after food contact with simulant liquids, as also seen for gas barrier permeability.

The interaction with polar liquids was evidenced by a slight increase in the –OH band intensity that was similar in all samples. These results highlighted the suitability of these materials to be used in humid environments as well as in contact with food. Furthermore, a molecular weight decrease of no more than 1–2% was recorded for all samples after stressed treatment, confirming the stability of such materials.

Migration tests need to be performed to evaluate the effectiveness of such materials to be placed in contact with real food. 

#### 3.3.2. Color Evaluation 

Film transparency and color are important requisites, especially if the material is used for food packaging application. Food color, associated with a high amount of naturally present pigments, has been always considered one of the key factors for evaluating food quality and taste, especially from the final consumers. Therefore, as previously reported [44], packaging should interfere as little as possible with the color of the food product, in order to preserve the consumer attractiveness. In Table 7, the film surface color determination for PPCE and P(PCExNCEy) samples are reported and compared to a white standard. 

On the CIE Lab Color scale, the lightness coefficient (L*) ranges from black (0) to white (100). For any L* value, the coordinates a* and b* situate the color on a rectangular coordinate grid perpendicular to the L* axis. At the origin (a* = 0 and b* = 0) the color is achromatic (gray). Moving on the horizontal axis, a positive a* value indicates a hue of red-purple and a negative a* value indicates a green hue. Moving on the vertical axis, a positive b* value indicates a yellow hue and a negative b* value indicates a blue hue [45]. P(PCExNCEy) films showed an L* closer to white, whereas a* and b* indicated a faint tendency toward a yellowish color (*h*_ab_ over 90°), like the as-synthesized polymer powder. A very low C* was recorded, meaning low color saturation and consequently a good transparency of the film, despite some differences being recorded related to the copolymer composition. The same characteristics were observed after treatments, indicating the good stability of the samples.

## 4. Conclusions

A new class of aliphatic biobased polyesters, previously synthesized and characterized from the thermal and mechanical points of view, was subjected to studies aiming to evaluate their barrier performances.

The results obtained are extremely interesting as the copolymers under investigation could be considered good candidates for food packaging application using the modified atmosphere packaging technique (MAP). The introduction of a neopentyl glycol unit into the PPCE did not result in a significant worsening of barrier performance with respect oxygen and nitrogen. As oxygen promotes the oxidation process, with subsequent deterioration of the chemical-physical, organoleptic, and quality properties of the packed food, a low oxygen permeation value can be considered a good result. Conversely, low permeation of nitrogen is a guarantee of package stability, avoiding bag collapses. In the case of the larger and polar CO_2_ molecules, a worsening in barrier performance due to copolymerization was found; the two side methyl groups present in the macromolecular chains rendered the polymer less polar and therefore decreased the solubility of carbon dioxide in the polymer matrix. However, an atmosphere poor in oxygen and rich in carbon dioxide decreases the metabolism of packed products or the spoilage activity, maintaining and/or prolonging the desired food shelf-life. 

A general worsening in the gas barrier properties after measurement in different moisture environments was recorded, showing an important interaction between the polymer matrix and water. On the contrary, all the samples under investigation showed good stability after food simulant contact.

In conclusion, due to their bio-based and biodegradable nature, the new investigated polyesters can be considered good candidates for substitution of the traditional petroleum-based polymers for packaging application.

## Figures and Tables

**Table 1:** Molecular, thermal, diffractometric, and mechanical characterization data of poly(propylene 1,4-cyclohexanedicarboxylate) (PPCE) and neopentyl glycol sub-units (P(PCExNCEy)) random copolyesters [12].

Polymer	*M*_n_^a^	*D*_index_^b^	NCE ^c^ (mol %)	Thickness (μm)	*T*_m_^d^ (°C)	*T*_g_^e^ (°C)	Χ_c_^d^ (%)
PPCE	36,398	2.2	0	246 ± 22	148	9	29 ± 4
P(PCE95NCE5)	29,549	2.9	5	292 ± 31	142	11	26 ± 3
P(PCE90NCE10)	31,124	2.2	10	268 ± 18	135	12	25 ± 2
P(PCE85NCE15)	27,522	2.6	15	238 ± 33	125	13	25 ± 2
P(PCE80NCE20)	25,386	2.4	20	308 ± 10	119	13	24 ± 2

Note: ^a^ number average molecular weight calculated by GPC analysis; ^b^ polydispersity index (D_index_) calculated by GPC analysis; ^c^ experimental copolymer composition calculated by ^1^H NMR; ^d^ from differential scanning calorimetry, first scan; and ^e^ from differential scanning calorimetry, second scan.

**Table 2:** GTR, S, D, and *t*_L_ data for the carbon dioxide (CO_2_) gas test and perm-selectivity ratio of the films.

Sample	GTR (cm^3^·cm/m^2^·d·atm)	*S* (cm^3^/cm^2^·atm)	*D* (cm^3^/s)	*t*_L_ (s)	CO_2_/O_2_	CO_2_/N_2_	O_2_/N_2_
PPCE	11.04	3.02 ± 4.04·E^-02^	4.18·E^-09^ ± 0.16·E^-11^	8513 ± 86	1.73	3.53	2.04
P(PCE95NCE5)	34.35	8.44·E^-01^ ± 4.04·E^-03^	4.64·E^-08^ ± 1.15·E^-10^	4623 ± 15	3.80	8.26	2.17
P(PCE90NCE10)	29.36	1.69 ± 1.0·E^-02^	1.98·E^-08^ ± 1.15·E^-10^	3464 ± 18	4.41	6.88	1.56
P(PCE85NCE15)	31.25	7.48·E^-01^ ± 1.60·E^-02^	4.77·E^-08^ ± 1.05·E^-09^	3312 ± 73	3.58	7.59	2.12
P(PCE80NCE20)	27.47	1.22 ± 3.51·E^-02^	2.57·E^-08^ ± 6.51·E^-10^	6043 ± 145	3.70	7.78	2.10

**Table 3:** *E*_GTR_, *H*_S_, and *E*_D_ data for CO_2_ pure gas, in the 8–38 °C temperature range, with the linear regression coefficients R^2^ provided in brackets.

Sample	PPCE	P(PCE95NCE5)	P(PCE90NCE10)	P(PCE85NCE15)	P(PCE80NCE20)
*E*_GTR_ (KJ/mol)	30.7 ± 0.13 (0.97)	26.8 ± 0.18 (0.97)	28.9 ± 0.11 (0.97)	30.1 ± 0.14 (0.99)	32.5 ± 0.21 (0.97)
*H*_S_ (KJ/mol)	-	−15.4 ± 0.11 (0.60)	−35.8 ± 0.16 (0.18)	5.7 ± 0.11 (0.02)	−7.24 ± 0.12 (0.01)
*E*_D_ (KJ/mol)	-	553 ± 0.18 (0.88)	8.32 ± 0.12 (0.00)	95.1 ± 0.15 (0.97)	388 ± 0.11 (0.15)

**Table 4:** Percentage increase (+) or decrease (–) in GTR for PPCE and P(PCExNCEy) samples under different ambient moistures.

Sample	CO_2_			
	23 °C 85% RH (KCl)	38 °C 90% RH (KNO_3_)	23 °C 85% RH (KCl)	38 °C 90% RH (KNO_3_)
PPCE	−22%	+136%	+7%	+54%
P(PCE95NCE5)	+126%	+23%	+11%	+62%
P(PCE90NCE10)	+85%	+197%	+5%	+47%
P(PCE85NCE15)	+55%	+159%	+8%	+35%
P(PCE80NCE20)	+53%	+121%	-22%	+60%

**Table 5:** Percentage (%) of CO_2_-GTR/O_2_-GTR increase (+) or decrease (−) for PPCE and P(PCExNCEy) copolymers after food simulant contact.

Sample/Simulant	Simulant A	Simulant B	Simulant C	Simulant D1
	CO_2_/O_2_			
PPCE	+8%/−21%	+24%/−18%	+25%/−17%	+37%/−28%
P(PCE95NCE5)	−70%/−20%	−64%/−17%	−50%/−10%	−34%/+4%
P(PCE90NCE10)	−36%/−3%	−47%/+11%	−36%/−5%	−36%/−1%
P(PCE85NCE15)	−49%/−25%	−49%/−24%	−37%/−17%	−34%/−8%
P(PCE80NCE20)	−31%/−10%	−15%/+6%	−3%/+11%	−20%/−13%

**Table 6:** Fourier transform infrared (FTIR) data for PPCE and P(PCExNCEy) films.

Chemical Group	Peak Position (cm^−1^)
–OH stretch (free)	3578
CH-stretch (of CH_2_)	2916 (ν_as_ CH_2_), 2853 (ν_s_ CH_2_)
–CH_3_ (pendant group)	2871 (ν_s_)
–C=O normal carbonyl stretch	1712
–CH-deformation symmetric and asymmetric bending	1472 (δ_s_ CH_2_)
C–O–H in-plane bend	1424
–CH_3_	1451 (δ_as_), 1378 (δ_s_)
–CH_2_-scissoring	1438
–C=O bending	1245
–C–O stretching	1178, 1153
–OH bending	1046
–CH_2_ wagging and twisting	1243, 1180
–CH_2_ rocking	731
O–H out-of-plane	992 (as), 945(s)
C–C stretch	920, 809

**Table 7:** Lightness coefficient (L*), a*, and b*, total color difference (Δ*E*), C* and *h*_ab_ of PPCE film and P(PCExNCEy) films.

Sample	L*	a*	b*	Δ*E*	C*	*h*_ab_
White standard	66.47 ± 0.01	−0.73 ± 0.01	1.22 ± 0	-	1.42	121
PPCE	63.67 ± 0.14	−0.89 ± 0.03	1.78 ± 0.13	2.85	1.99	139
P(PCE95NCE5)	61.86 ± 0.69	−0.88 ± 0.02	2.93 ± 0.52	4.92	3.06	107
P(PCE90NCE10)	63.49 ± 0.60	−0.87 ± 0.02	1.85 ± 0.28	3.05	2.04	115
P(PCE85NCE15)	62.92 ± 0.32	−0.95 ± 0.05	2.52 ± 0.27	3.79	2.69	111
P(PCE80NCE20)	61.96 ± 0.43	−0.98 ± 0.02	2.88 ± 0.24	4.81	3.04	109

*h*_ab_ = 0°, red-purple; *h*_ab_ = 90°, yellow; *h*_ab_ = 180°, green; *h*_ab_ = 270°, blue.
